# Correlation Between *Cryptococcus* Infection and the Nasal Mycobiota in a Population of Free-Ranging Koalas (*Phascolarctos cinereus*) in New South Wales, Australia

**DOI:** 10.3390/jof11010064

**Published:** 2025-01-15

**Authors:** Andrew S. McPherson, Sophie L. Haworth, Alex Kan, Luisa Monteiro de Miranda, Mark B. Krockenberger

**Affiliations:** Sydney School of Veterinary Science, Faculty of Science, The University of Sydney, Sydney, NSW 2006, Australia; andrewmcpherson787@gmail.com (A.S.M.); sophie.haworth@hotmail.com (S.L.H.); lxkan96@gmail.com (A.K.); luisa.miranda@sydney.edu.au (L.M.d.M.)

**Keywords:** koala, *Cryptococcus*, mycobiome, ITS

## Abstract

Cryptococcosis is a fungal disease in humans and animals, caused by the *Cryptococcus neoformans* and *Cryptococcus gattii* species complexes. Clinical cryptococcosis primarily manifests as upper respiratory tract disease; however, dissemination to other organs, particularly the brain, can occur. Nasal colonisation and subclinical cryptococcosis are common in koalas (*Phascolarctos cinereus*) due to their shared environmental niche with *Cryptococcus*: *Eucalyptus* trees. However, for reasons that remain unclear, the prevalence of clinical disease is low in koalas. Interactions between respiratory pathogens and the nasal mycobiome are thought to play a role in the development and progression of numerous respiratory diseases. As such, this study aimed to characterise the mycobiome of the nasal vestibule in koalas with and without evidence of cryptococcal colonisation and subclinical disease via the next-generation sequencing (NGS) of the ITS1 region of the fungal internal transcribed spacer (ITS) gene. Samples were collected from 47 koalas from a population of free-ranging koalas in the Liverpool Plains, NSW, Australia, with a known history of *Cryptococcus* exposure and nasal colonisation. Of the 47 animals tested, 6.4% were culture-positive only, 4.3% were seropositive only, and 2.1% were culture- and seropositive. *C. gattii* was detected in four samples via NGS. *C. neoformans* was not detected via NGS. There were no significant differences in the nasal mycobiomes of *Cryptococcus*-positive and -negative animals; thus, we could not establish a definitive association between the mycobiome and infection outcomes. We identified a number of fungal genera that were significantly more abundant in samples from *Cryptococcus*-positive animals, but there was no apparent relationship between these genera and the development of cryptococcosis. This study represents the first investigation of the nasal mycobiota of wild koalas. Further studies involving koalas with clinical disease are necessary to determine the role of the nasal mycobiota in the development of cryptococcosis.

## 1. Introduction

Cryptococcosis is a fungal disease in humans and animals, caused by the *Cryptococcus neoformans* and *Cryptococcus gattii* species complexes [1,2]. Contact with *Cryptococcus* species complexes organisms occurs largely via the inhalation of desiccated yeast cells or basidiospores from the environment [3,4,5], resulting in the colonisation of the respiratory tract, which has been reported in livestock, wildlife, companion animals, and humans [1,6,7,8,9]. The interaction between *Cryptococcus* species complexes organisms and the host can develop further into subclinical disease in humans and animals, which is detected through the measurement of cryptococcal antigens in the blood (antigenaemia) in the absence of clinical disease [10,11,12,13,14]. If the interaction between the potential pathogen and host develops further, the clinical disease initially primarily affects the respiratory tract [2,8], with dissemination to other organs as disease progression occurs, particularly to the brain, causing neurological manifestations such as meningoencephalitis and ultimately the death of the animal [2,8,11].

The koala (*Phascolarctos cinereus*) is an iconic Australian arboreal marsupial species that is in decline in some parts of Australia due to habitat loss and fragmentation [15,16], natural disasters [17], and infectious diseases [18,19]. Nasal colonisation and subclinical cryptococcosis is common in koalas, with the colonisation of the nasal cavity reported in up to 100% of animals in a captive population of koalas in near Coffs Harbour, NSW [4]; 35% in a population near Port Macquarie, NSW [4]; and 6.6% in a population on the Liverpool Plains, NSW [20]. The prevalence of clinical cryptococcosis varies between populations and studies, likely approximately 3 to 4% of koalas [21]. When it occurs, it is an important cause of respiratory tract and neurological disease [8]. The high prevalence of clinical and subclinical disease in koalas is a consequence of the environmental niche shared by koalas and the *Cryptococcus gattii* species complex, namely *Eucalyptus* trees, which koalas utilise for shelter and as a primary food source; these are the primary environmental niche of the *Cryptococcus gattii* species complex in Australia [4,22,23,24]. Cryptococcosis in koalas is mostly associated with infection by *C. gattii* [8,20], of which there are four main molecular types: VGI, VGII, VGIII, and VGIV [25]. Of these, VGI is most often identified in samples from the environment and koalas [5,23,26].

The pathogenesis of clinical cryptococcosis in koalas is complex and not fully understood; however, the high prevalence of nasal colonisation and subclinical disease [6,27] only translates into a much lower prevalence of clinical disease, suggesting that environmental exposure to *Cryptococcus* alone does not lead to cryptococcosis. However, there is a strong association between the environmental *Cryptococcus* load, which varies between trees and locations [4,23], and nasal colonisation in koalas [4,22,27]. Environmental “hot spots”, in which the environmental *Cryptococcus* loads are high, are regarded as a key factor in the epidemiology of cryptococcosis in koalas [4,22,27]. The significance of host and environmental factors such as age, gender, the environmental *Cryptococcus* load, and the home range in the epidemiology of cryptococcosis in koalas have also been investigated [4,6,11,20,22,27], but few consistent associations have been identified across studies.

The role of the respiratory tract microbiome in the development, progression, and persistence of respiratory diseases has been described in humans and animals [28,29,30,31]. The respiratory tract microbiome is diverse and contains bacteria, fungi, archaea, and viruses. To date, the bacterial community of the respiratory tract has received the greatest attention. The role of the fungal community (mycobiome) of the respiratory tract has received comparatively little attention. However, there is evidence that the mycobiome has a significant impact on the development and progression of both infectious and non-infectious respiratory diseases, including allergic rhinitis [32,33], asthma [34], cystic fibrosis [35], and COVID-19 [36]. To date, the nasal mycobiome of the koala has not yet been characterised, and its association with *Cryptococcus gattii* species complex colonisation and cryptococcal antigenaemia is unknown. The aim of this study was to characterise and compare the compositions of the mycobiota of the nasal vestibule in koalas with and without evidence of cryptococcal colonisation and subclinical disease.

## 2. Materials and Methods

### 2.1. Animal Ethics Statement

All protocols were approved by The University of Sydney’s Animal Ethics Committee (project no. 2019/0955).

### 2.2. Sample Collection

Samples were collected in January 2019 (16 January 2019 to 24 January 2019) from a population of free-ranging koalas in the Liverpool Plains near Gunnedah, NSW, Australia. This population of koalas has been studied in detail from 2015, with a known history of *Cryptococcus* exposure and nasal colonisation [37]. This population was identified as an expanding koala population in the early 2000s, with a subsequent reduction in numbers [38]. Koalas were visually identified by investigators, captured by the ‘flag and pole’ technique [39], and restrained for sedation. Each koala was sedated with alfaxalone (Alfaxan; Jurox Pty Ltd., Rutherford, NSW, Australia) at 1.8 mg/kg, injected intramuscularly into the quadriceps muscle. Upon sedation, each animal was physically examined, and diagnostic specimens were collected. Blood (3 mL) was collected from the cephalic vein into plain tubes and transported to the laboratory while cooled. Nasal swabs were collected from each animal by inserting a sterile swab (Interpath Services, Somerton, VIC, Australia) approximately 2–3 cm into the left and right nasal cavity and rolling the head of the swab across the surface of the mucosa. Two swabs were collected from each animal: a dry sterile cotton swab (Interpath Services, Somerton, VIC, Australia) for DNA extraction, which was placed into a 1.8 mL cryovial and flash-frozen in liquid nitrogen for transport to the laboratory, and a wet cotton swab for fungal culture, transported in Amies medium without charcoal (Transystem; Copan, Murrieta, CA, USA) in a portable fridge at 4 °C.

### 2.3. Serology

Blood samples were processed within 12 h of collection. Blood samples in plain vacutainers were centrifuged at 5000 rpm for 5 min and the serum was collected and transferred to a plain cryotube. Serum samples were stored in liquid nitrogen and then transferred to a −80 °C freezer prior to testing. Each serum sample was tested with a cryptococcal antigen lateral flow assay (LFA) (CrAg LFA; IMMY, Norman, OK, USA) according to the manufacturer’s instructions.

### 2.4. Cryptococcus Species Complex Culture

Nasal swabs were removed from the Amies medium and streaked onto bird seed agar (BSA), a selective medium for the isolation of *Cryptococcus* [40]. Plates were incubated aerobically at 28 °C for three days before being examined. *Cryptococcus* colonies were identified by their morphology, as described previously [41]. Putative *Cryptococcus* colonies were picked and sub-cultured onto Sabouraud’s dextrose agar (SDA) and incubated at 37 °C for three days. Isolates were sub-cultured onto SDA up to five more times until a pure culture of each isolate was obtained. Once a pure culture was obtained, the isolate was sub-cultured onto BSA again and incubated as described above to confirm the colony morphology and for MLST.

### 2.5. Multi-Locus Sequence Typing

Multi-locus sequence typing (MLST) of putative *Cryptococcus* species complex isolates was performed by the Medical Mycology Research Laboratory at the Westmead Institute, as described previously [20,42], to determine the species and molecular type of each *Cryptococcus* isolate.

### 2.6. DNA Extraction, NGS, and Bioinformatics

Swabs collected for DNA extraction were sent to the Australian Genome Research Facility (AGRF; Brisbane, QLD, Australia) for DNA extraction, PCR amplification of the ITS1 region, library preparation, and NGS. DNA was extracted with the Powersoil Pro Kit (Qiagen, Hilden, Germany) according to the manufacturer’s instructions. The ITS1 region of the fungal internal transcribed spacer (ITS) gene was amplified using the primers ITS1F (5′-CTTGGTCATTTAGAGGAAGTAA-3′) and ITS2 (5′-GCTGCGTTCTTCATCGATGC-3′). NGS was performed on an Illumina MiSeq (Illumina, San Diego, CA, USA) with 300 bp (bp) paired-end (PE) chemistry. Bioinformatics was performed with QIIME2 v2020.6 [43], through the University of Sydney’s high-performance (HPC) computing system. Primer and adapter trimming were performed with the QIIME2 cutadapt plugin with the --p-match-adapter-wildcards, --p-match-read-wildcards, and --p-discard-untrimmed flags. Denoising was performed with the QIIME2 DADA2 plugin [44]. Twenty bp were trimmed from the 5′ end of the forward and reverse reads, respectively. Quality trimming of the 3′ end of the reads was performed using the default parameters and the option --p-pooling-method = pseudo. Reads were then classified into amplicon sequence variants (ASVs, or features) based on a minimum of 99% sequence similarity. Taxonomy was assigned to the representative sequences for each ASV using a Naïve Bayes classifier trained on a hybrid database containing full-length ITS sequences from the UNITE database v13-9 developer set [45] and all full-length *Cryptococcus* spp. ITS sequences available in the International Society for Human and Animal Mycology (ISHAM) barcoding database [46]. ASVs classified as chloroplast, mitochondrial, archaea, bacterial, or “unclassified” at the kingdom level were filtered from the dataset. An alignment of the representative sequences for each ASV was generated with MAFFT, and a phylogenetic tree was generated with FastTree, using the QIIME2 phylogeny plugin.

### 2.7. Statistical Analysis

Statistical analysis of the NGS count data was performed in RStudio v2023.06.0. Alpha diversity was calculated for each sample using the metrics of the observed features, Shannon’s diversity index, Simpson’s diversity index, and the inverse Simpson’s diversity index with the R package phyloseq v1.44.0 [47]. The count data were rarefied to a depth of 19,220 reads per sample, and alpha rarefaction curves were generated and inspected to ensure that this depth was sufficient to capture the true level of community diversity in each sample. Differences in alpha diversity between *Cryptococcus*-positive and -negative animals were analysed with a Wilcoxon rank sum test. Beta diversity analyses were also performed to evaluate differences in the nasal microbiota of *Cryptococcus*-positive and -negative animals. Count data were centred log ratio (clr)-transformed with the R package microbiome v1.22.0. Distance matrices were generated using Aitchison distances within the R package phyloseq and analysed using the “ADONIS” function within the R package vegan v2.6.4 [48]. A significant *p*-value of 0.05 was used for all alpha and beta diversity analyses.

### 2.8. Differential Abundance Analysis

Differential abundance analysis was performed at the genus level with the R package DESeq2 v1.40.2 [49] to identify differentially abundant ASVs in animals that were classified as *Cryptococcus*-positive or -negative with each respective test. Differences between *Cryptococcus*-positive and -negative animals were determined using a Wald test with a parametric fit of dispersions and Benjamini–Hochberg-adjusted *p*-values. ASVs with an adjusted *p*-value of <0.01 were considered significant. In each analysis, negative samples were used as a reference category.

## 3. Results

### 3.1. Sample Collection

Samples were collected from 47 koalas—18 males and 29 females—predominantly with a poor to moderate body condition score, with a spread across all age classes but skewed towards older mature adults.

### 3.2. Detection of Cryptococcus

Colonies with brown colouration were observed on 6/47 (12.8%) primary cultures on BSA, and four of these could be purified on SDA. The four isolates were subjected to MLST for species identification and molecular typing, and all were identified as *C. gattii* molecular type VGI. *Cryptococcus* antigen was detected in 3/47 (6.4%) of serum samples tested with the CrAg LFA test (Table 1). Of the 47 animals tested, 6.4% (3/47) were culture-positive only, 4.3% (2/47) were CrAg-LFA-positive only, 2.1% (1/47) were culture- and CrAg-LFA-positive, and 87% (41/47) were culture- and LFA-negative (Table 1).

ASVs classified as *C. gattii* were identified in 4/38 (10.5%) samples that were sent for sequencing. Of these, one was obtained from an animal that was culture-positive only, two were obtained from animals that were LFA-positive only, and one was obtained from an animal that was both culture- and LFA-positive (Table 1). *C. gattii* was not detected in a sample from one animal that was culture-positive only (Table 1). The relative abundance of *C. gattii* in these samples ranged from 0.12 to 3.19% of the total community (Table 1). The highest relative abundance (3.19%) was for a sample collected from an animal that was LFA-positive but culture-negative (Table 1).

### 3.3. Characterisation of the Nasal Mycobiota

A total of 47 nasal swabs were submitted to AGRF for DNA extraction, PCR amplification, and sequencing of the ITS1 region of the fungal ITS gene. Nine samples did not pass quality control as they did not contain a sufficient concentration of DNA (0.20 ng DNA/µL) for PCR amplification. The remaining 38 samples passed quality control and proceeded to PCR amplification, library preparation, and sequencing. A total of 4,688,309 reads were obtained from the 38 samples, with an average of 123,377 reads obtained per sample (range: 41,750–189,254) (Supplementary Table S1). After filtering, a total of 2,606,507 reads remained, with an average of 68,592 reads per sample (range: 19,226–127,419) (Supplementary Table S1).

### 3.4. Diversity Analyses

**Figure 1:**
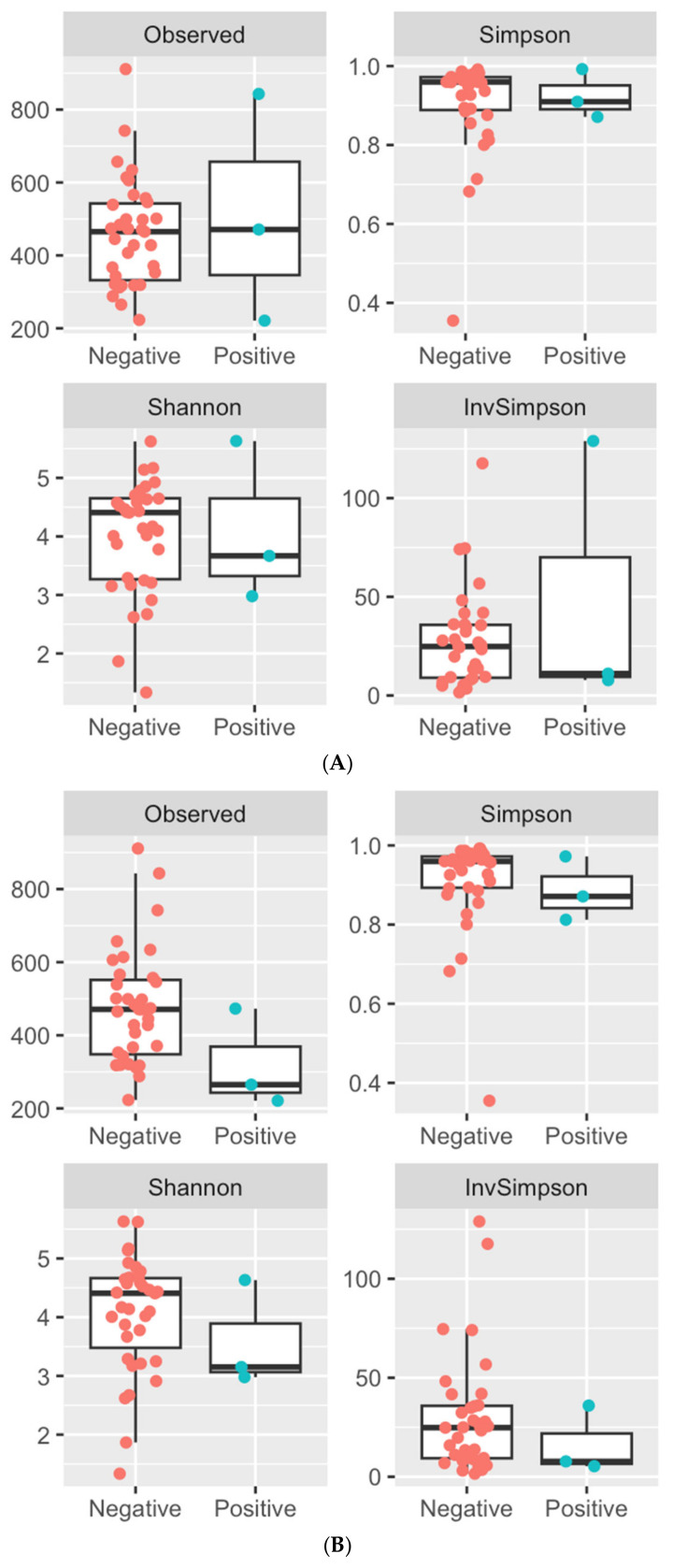

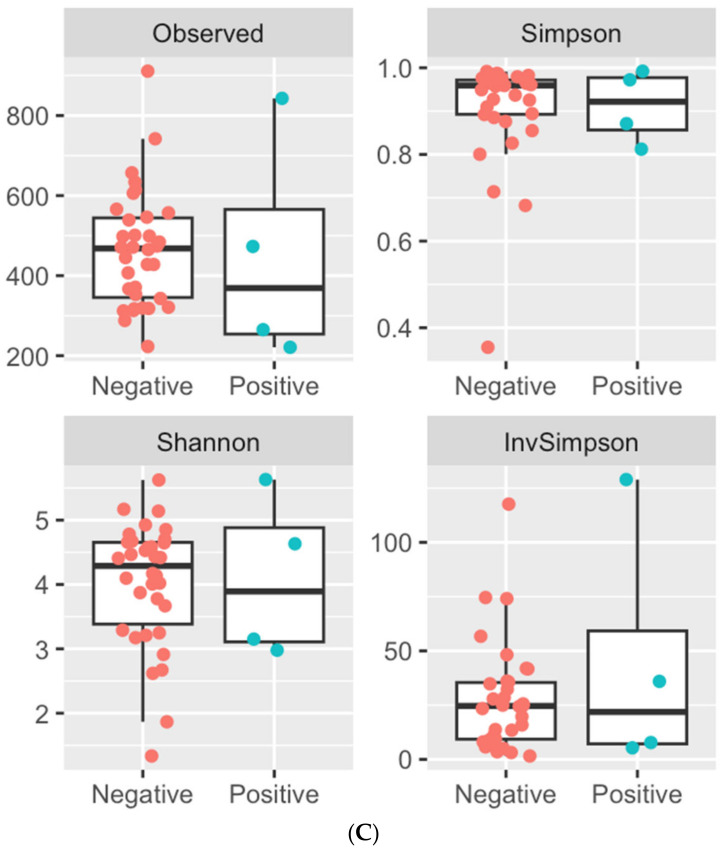
The mycobiota of the nasal vestibule was characterised in 47 koalas from a population on the Liverpool Plains, NSW, Australia. The diversity of the fungal communities was evaluated using four alpha diversity metrics: number of observed features, Simpson’s diversity index, Shannon’s diversity index, and inverse Simpson’s index. Alpha diversity boxplots for animals that were *Cryptococcus*-positive and -negative according to (**A**) culture, (**B**) LFA, and (**C**) NGS are presented.

Alpha and beta diversity metrics were used to compare the diversity of the fungal communities in samples from animals that were classified as *Cryptococcus*-positive or -negative by culture, LFA, or NGS. There was no significant difference in alpha diversity between samples classified as *Cryptococcus*-positive or -negative via any test (Figure 1, Supplementary Table S2). To analyse beta diversity, the count data were centred log ratio (clr)-transformed, and distance matrices were generated based on the Aitchison distances and analysed using ADONIS. There were no significant differences in beta diversity between animals classified as *Cryptococcus*-positive or -negative via culture (*p* = 0.359), LFA (*p* = 0.789), or NGS (*p* = 0.211).

### 3.5. Taxonomic Analysis

**Figure 2:**
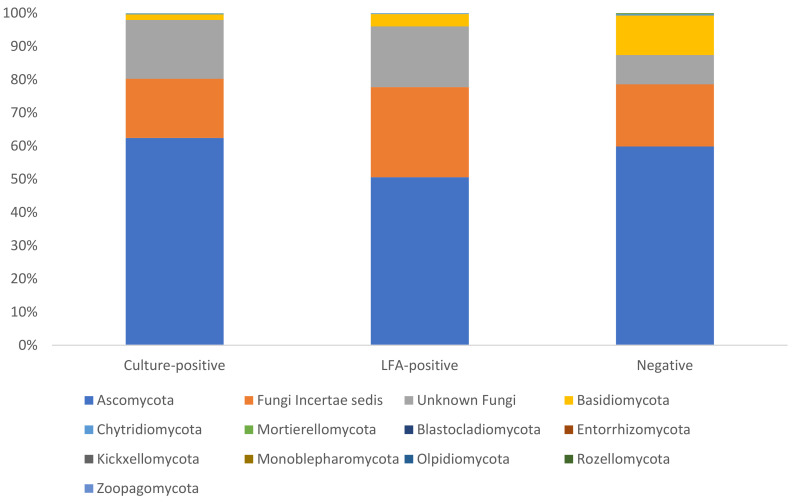
Relative abundance of fungal phyla identified in samples from koalas that were *Cryptococcus* culture-positive, CrAgLFA-positive, or negative by both tests.

**Figure 3:**
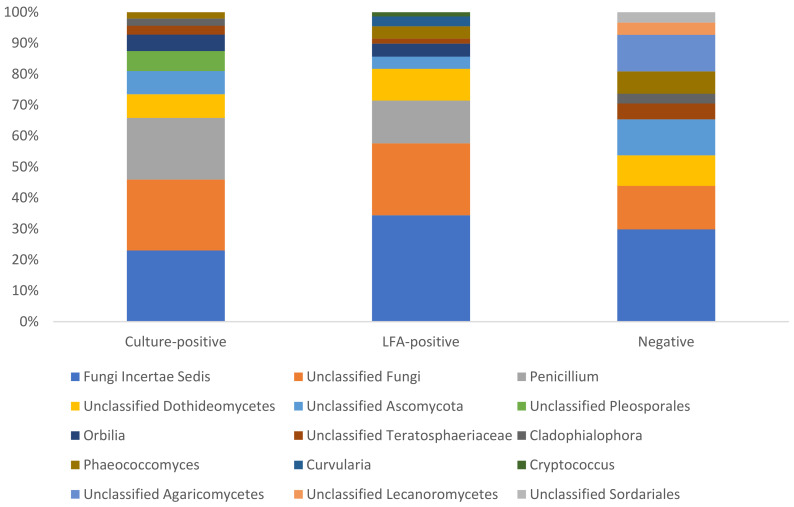
The ten most abundant fungal genera identified in samples from koalas that were *Cryptococcus* culture-positive, CrAgLFA-positive, or negative by both tests.

The composition of the mycobiota was similar across samples, irrespective of the *Cryptococcus* status of the animal from which it was collected. The taxonomy of approximately 30% to 50% of the ASVs in each sample could not be resolved beyond the kingdom level. The phylum Ascomycota was the most abundant phylum across all samples, representing an average of approximately 50% to 60% of the fungal community in each sample (Figure 2). Few of the dominant taxa in each sample could be classified to the genus level. There were some exceptions to this, however, with *Penicillium* abundant in samples from culture-positive (15.5%), LFA-positive (10.9%), and NGS-positive (9.4%) animals and *Phaeococcomyces* in samples from culture-negative (4.46%), LFA-negative (4.35%), and NGS-negative (6%) animals (Figure 3).

### 3.6. Differential Abundance Analysis

Differential abundance analyses were undertaken using DeSeq2 to identify differentially abundant ASVs in samples from animals that were *Cryptococcus*-positive or -negative according to culture, LFA, or NGS. The results of these analyses are provided in Supplementary File S1. In each analysis, negative samples were used as a reference category, i.e., fold changes refer to a higher or lower relative abundance of each ASV in *Cryptococcus*-positive animals in comparison to *Cryptococcus*-negative animals. In each analysis, the taxonomy of most differentially abundant ASVs could not be resolved beyond the kingdom or phylum levels (Supplementary File S1). A total of 119 ASVs were differentially abundant between animals that were culture-positive and culture-negative (Supplementary File S1). Of those that could be classified at the genus level, the smallest adjusted *p*-values were for ASVs identified as *Pyrenochaeta* (*p*.adj = 2.73 × 10^−5^; log2 fold change = 25.6) and *Neocucurbitaria* (*p*.adj = 1.83 × 10^−5^; log2 fold change = 26.6). A total of 141 ASVs were differentially abundant between animals that were LFA-positive and LFA-negative (Supplementary File S1). Of those that could be classified at the genus level, the smallest adjusted *p*-value was for an ASV identified as *Neocucurbitaria* (*p*.adj = 1.76 × 10^−5^; log2 fold change = 26.3). A total of 98 features were differentially abundant between animals that were NGS-positive and NGS-negative (Supplementary File S1). Of those that could be classified at the genus level, the smallest adjusted *p*-values were for ASVs identified as *Mortierella* (*p*.adj = 5.55 × 10^−5^; log2 fold change = −29.5), *Orbilia* (*p*.adj = 8.80 × 10^−8^; log2 fold change = 22.8), and *Filobasidium* (*p*.adj = 5.55 × 10^−5^; log2 fold change = 21.1). *Cryptococcus* was identified as differentially abundant in samples from culture-positive (*p*.adj = 1.72 × 10^−5^; log2 fold change = 26.9), LFA-positive (*p*.adj = 2.09 × 10^−5^; log2 fold change = 26.0), and NGS-positive (*p*.adj = 0.001; log2 fold change = 22.7) animals.

## 4. Discussion

The progression of the host–pathogen interaction between koalas and organisms of the *C. neoformans* and *C. gattii* species complexes from contact, colonisation, through limited reversible tissue invasion (subclinical disease) to clinical disease, is of great interest with regards the pathogenesis of cryptococcosis in all species. Colonisation of the nasal mucosa by the fungal pathogen *Cryptococcus* has been documented in up to 100% of koalas in some populations [4,6,8,11,27]. However, colonisation does not always lead to invasive infections or clinical cryptococcosis, and persistent subclinical (asymptomatic) cryptococcosis is also common in koalas [4,6,8,11,27]. The role of the respiratory tract mycobiome in the development of cryptococcosis in koalas has not previously been investigated, and this study represents the first time that the composition of the nasal mycobiota has been described in this species.

In the present study, the prevalence of nasal colonisation and antigenaemia was 8.5% (4/47) and 6.4% (3/47), respectively. Four *Cryptococcus* isolates were obtained in the present study, all of which were classified as *C. gattii* VGI by MLST. ASVs classified as *C. gattii* were also identified in samples from four animals that were culture-positive or antigenemic. The species *C. neoformans* was not identified in any samples by any test. This result is consistent with previous studies, which have demonstrated that *C. gattii* VGI is the most common molecular type identified in koalas in NSW [5]. This species is also detected most often in environmental samples in NSW [4,23,24], so koalas are most likely to be exposed to this molecular type. Cryptococcosis in koalas is most commonly caused by isolates of the *C. gattii* species complex (Krockenberger et al. 2003), with only one reported case caused by the *C. neoformans* species complex [50]. The results of this study confirm the significance of this molecular type to the epidemiology of cryptococcosis in NSW koala populations.

The results of the three methods of detection (culture, LFA, NGS) used in this study were not completely concordant. The discrepancies between the three tests reflect the complex aetiopathogenesis of *Cryptococcus* infection in koalas and the difficulty of identifying infected animals at different stages of interaction between the koala and the potential pathogen. We cannot exclude the possibility that PCR bias may have had an impact on the NGS dataset, and, although there was an overall good correlation with the *Cryptococcus* culture and serology results, future studies may benefit from the inclusion of a mock fungal community as a positive control during DNA extraction and NGS. Culture is the gold standard for the detection of *Cryptococcus* and is the most widely used method of detection in diagnostic laboratories [51]. However, culture is complicated in specimens containing an abundance of fast-growing environmental hyphal fungi, which reduce the detectability of the slower-growing pathogenic *Cryptococcus* species complexes isolates. The use of selective media, such as BSA, can enhance the sensitivity of culture by making it easier to identify *Cryptococcus* colonies and limiting the growth of other fungi [41]. Benomyl birdseed agar could be considered in selectively isolating *Cryptococcus* from specimens expected to be rich in mould-forming environmental fungi and may be an alternative to minimise this issue in future studies. Considering that *Cryptococcus* nasal colonisation may or may not progress to tissue invasion and consequent antigenemia, the clinical relevance of the nasal colonisation is debatable in koalas, as this finding alone does not prompt any clinical action. Thus, if the *Cryptococcus* abundance is not high enough to grow in culture, then its presence is not likely clinically relevant. Nonetheless, a negative culture in the nasal cavity does not preclude an interaction between the host and the potential pathogen, as sampling may not represent the nasal mucosal colonisation accurately and colonisation can be intermittent in some animals. Altogether, these features indicate a complex interaction between koalas, the nasal cavity mycobiome, and isolates of the pathogenic *Cryptococcus* species complexes and highlight the necessity of carefully considering the sample type and diagnostic technique when interpreting the results.

Broadly, the composition of the mycobiota was similar in *Cryptococcus*-positive and -negative animals at the community level. The phylum Ascomycota was the most abundant phylum across the 38 samples sequenced in this study, representing 50% to 60% of the total community. The majority of the species in this phylum are spore-forming and ubiquitous in the environment; hence, it has been reported as the most abundant fungal phylum in the respiratory tracts of humans and animals previously [52,53,54,55,56]. This phylum was also reported to be the most abundant phylum in *Eucalyptus* tree hollows [24]. Differential abundance analyses were performed to identify differentially abundant fungal ASVs between animals that were *Cryptococcus*-positive and *Cryptococcus*-negative according to each of the three tests (Supplementary File S1). The majority of the differentially abundant ASVs identified in these analyses could not be classified beyond the Order or Family levels (Supplementary File S1). Of those that could be classified to the genus level, most were common environmental fungi of little clinical significance. However, some of the genera identified are known or emerging opportunistic pathogens that have been associated with infections in humans and animals. The genera *Pyrenochaeta* (*p*.adj = 2.73 × 10^−5^; log2 fold change = 25.6) and *Neocucurbitaria* (*p*.adj = 1.83 × 10^−5^; log2 fold change = 26.6) were significantly more abundant in culture-positive animals than in culture-negative animals. The genus *Pyrenochaeta* is a soil-borne filamentous fungus that is most commonly known as a pathogen of tomato plants [57] but has been isolated from an ocular infection in a human in Spain [58], has been associated with skin infections in humans [59], and is increasingly associated with subcutaneous phaeohyphomycosis in humans [60,61,62]. The genus *Neocucurbitaria* is a coelomycetous fungus that has been associated with cases of fungal keratitis in humans [63,64]. The genus *Mortierella*, which was significantly more abundant in samples from NGS-negative animals than in NGS-positive animals, is ubiquitous in the environment, including *Eucalyptus* tree hollows [24]. This genus has been identified as a cause of meningoencephalitis [65], systemic mycosis [66,67], and abortion [13] in cattle; keratomycosis in a horse [68]; and disseminated pulmonary infection in a child [69]. The genus *Filobasidium* has been reported in rare cases of otomycosis [70] and meningitis [71]. Interestingly, some strains of the species *Filobasidium capsuligenum* are known to produce a killer toxin that has anti-cryptococcal activity [72,73], but this species was not identified in this study. With the exception of *Filobasidium*, there is no apparent relationship between these genera and *Cryptococcus*. Further investigation of the interactions between these genera and *Cryptococcus* is needed to identify what role, if any, they might play in the disease process.

A key challenge of marker gene surveys is the presence of taxa that cannot be identified beyond the kingdom level [45,74], particularly in environmental samples, where there may be a large abundance of fungi that have not been isolated and characterised in the laboratory. In the present study, approximately 30% of the ASVs identified in the dataset could only be identified to the kingdom level. With such limited information about these organisms, it is not possible to determine their veterinary significance. In this study, we used the UNITE database [45], a curated database that consists of approximately 1,000,000 full-length ITS sequences clustered into 459,000 species hypotheses, to assign taxonomy to the ASVs identified in the dataset. However, the ITS sequences included in the UNITE database represent a small proportion of the 2 to 11 million fungal species that are estimated to exist [75]. Other databases can be used, such as GenBank; however, many of these databases are not curated and contain sequences from studies that have not been through peer review. Therefore, taxonomic information obtained from such databases can be unreliable and needs to be interpreted with caution.

The association between *Cryptococcus* detection and non-fungal living organisms composing the nasal microbiota of koalas was not within the scope of this study, but it constitutes an important avenue for future research. The interaction between fungal and bacteria communities in the microbiota can impact fungal pathogenicity and modulate the host antifungal response, consequently impacting host health. *Cryptococcus*–bacteria interactions in the context of the host microbiota have been little explored, but in vitro studies have suggested that bacteria commonly found in the human microbiota or in the environment, including *Staphylococcus aureus, Klebsiella aerogenes*, and *Pseudomonas aeruginosa*, can impact, positively or negatively, *Cryptococcus* growth and physiology. Further studies are encouraged to identify the koala’s nasal bacterial microbiota and its potential impact in the *Cryptococcus*–koala interaction.

## 5. Conclusions

There were no significant differences in the nasal mycobiota of *Cryptococcus* culture-positive and -negative animals, so we could not establish a definitive association between the mycobiome and cryptococcal infection outcomes. Considering the limited ecological niche of koalas, along with the lack of variation in the nasal mycobiome of the animals in this study and the low frequency of cryptococcal disease in free-ranging koalas, it is likely that the nasal mycobiome is fairly uniform and well adapted; however, the relationship with the environmental mycobiome needs further investigation. Environmental changes and coinfections by other pathogens are factors to be further explored as potentially impacting the nasal microenvironment, mycobiome, and overall koala immunity. We identified a number of fungal genera that were significantly more abundant in samples from *Cryptococcus*-positive animals, but no apparent relationship between these genera and the development of cryptococcosis has been described previously. Lastly, although captive koalas were not the object of this study, the role of their nasal mycobiome in the development of respiratory conditions should be investigated and compared to that in wild koalas, as there is a much higher prevalence of cryptococcosis in captive koalas and different environmental conditions in comparison to koalas’ natural habitats. This study represents the first investigation of the nasal mycobiota of wild koalas, and future longitudinal studies would benefit from a longitudinal design to investigate temporal associations between the environment, *Cryptococcocus* colonisation, and the nasal mycobiota.

## Figures and Tables

**Table 1:** Test results for koalas identified as *Cryptococcus*-positive by culture, CrAg-LFA, or NGS. LFA = lateral flow assay. NGS = next-generation sequencing. NT = not tested. * Relative abundance refers to the proportion of the total fungal population that *C. gattii* represented according to the NGS data.

Sample ID	Culture	CrAg-LFA	NGS	Relative Abundance *
USYD006M	Positive	Negative	Negative	0
USYD014M	Positive	Positive	Positive	0.05
USYD017F	Negative	Positive	Positive	3.19
USYD069M	Negative	Positive	Positive	0.3
USYD076F	Positive	Negative	Positive	0.12
USYD053M	Positive	Negative	NT	NT

## Data Availability

The raw data supporting the conclusions of this article will be made available by the authors on request.

## Supplementary Materials

The following information can be downloaded at https://www.mdpi.com/article/10.3390/jof11010064/s1, Table S1: Differential abundance analysis (DeSeq2) to identify differentially abundant ASVs in samples from animals that were *Cryptococcus*-positive or -negative according to culture, LFA, or NGS. In each analysis, negative samples were used as a reference category, i.e., fold changes refer to a higher or lower relative abundance of each ASV in *Cryptococcus*-positive animals in comparison to *Cryptococcus*-negative animals. A total of 119 ASVs were differentially abundant between animals that were culture-positive and culture-negative
